# Selective Autophagy Receptor CsNBR1 Confers Citrus Huanglongbing Resistance by Degrading ‘*Candidatus* Liberibacter Asiaticus’ Virulence Effectors

**DOI:** 10.1111/mpp.70310

**Published:** 2026-06-29

**Authors:** Yaqian Shi, Xuejin Cui, Fang Fang, Zaiyu Yang, Yalin Mei, Shimian Ma, Changyong Zhou, Xuefeng Wang

**Affiliations:** ^1^ National Citrus Engineering Research Center, Citrus Research Institute Southwest University Chongqing China; ^2^ Integrative Science Center of Germplasm Creation in Western China (CHONGQING) Science City Southwest University Chongqing China; ^3^ Jiangxi Key Laboratory of Horticultural Crops (Fruit, Vegetable & Tea) Breeding Jiangxi Academy of Agricultural Sciences Nanchang China

**Keywords:** ‘*Candidatus* Liberibacter asiaticus’, breeding, CsNBR1, selective autophagy, virulence effectors

## Abstract

The selective autophagy receptor NEIGHBOUR OF BRCA1 (NBR1) is a key regulator of plant immunity, but its role against bacterial pathogens remains poorly understood. Here, we demonstrated that overexpression of *CsNBR1* confers citrus resistance to huanglongbing (HLB) caused by ‘*Candidatus* Liberibacter asiaticus’ (*C*Las). CsNBR1 can interact with multiple virulence effector proteins of *C*Las, including SDE5115, SDE1, SDE19 and SDE5, leading to their degradation via the autophagic pathway. In addition, expression of *CsNBR1* in *Nicotiana benthamiana* mitigated the leaf curling and dwarfing phenotypes triggered by SDE1 by reducing the protein content of SDE1. These findings reveal that CsNBR1 positively regulates citrus defence against *C*Las by degrading its effector proteins via selective autophagy, providing both mechanistic insights and a potential target for resistance breeding.

## Graphical abstract

Overexpression of *CsNBR1* confers HLB resistance in citrus by degrading *C*Las effector proteins, including SDE5115, SDE1, SDE19 and SDE5, via the autophagy pathway.

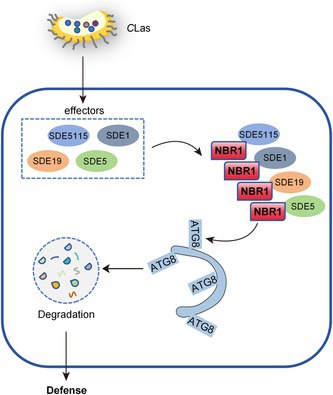

1

Citrus huanglongbing (HLB, also known as citrus greening) caused by the phloem‐colonizing bacterium ‘*Candidatus* Liberibacter asiaticus’ (*C*Las) is the world's most widespread citrus disease (Bastianel et al. 2005; Bové 2006). The *C*Las genome is approximately 1.23 Mb and lacks type III and type IV secretion systems, but it encodes a complete Sec‐dependent secretion (SEC) pathway, which translocates numerous SEC‐dependent effectors (SDEs) into host cells (Duan et al. 2009; Prasad et al. 2016). These SDEs are critical virulence determinants and can suppress host immune responses by targeting key defence regulators. For instance, SDE1 (CLIBASIA_05315) inhibits citrus papain‐like cysteine proteases (PLCPs) (Clark et al. 2018). SDE4405 (CLIBASIA_04405) directly binds to citrus ATG8 inducing autophagy (Shi, Yang, et al. 2023). SDE3 (CLIBASIA_00420) interacts with CsGAPC and disrupts CsATG8‐mediated autophagy (Shi, Gong, et al. 2023). SDE19 (CLIBASIA_05320) targets CsSec12, interfering with host vesicle trafficking and suppresses defence‐related protein secretion (Huang et al. 2024). SDE5 (CLIBASIA_02470) decreases jasmonic acid (JA) accumulation, enhancing host susceptibility to *C*Las (Zhao et al. 2025). Thus, identifying host factors that antagonize the action of these effectors is therefore essential for developing HLB resistance.

Selective autophagy plays a central role in plant innate immunity by removing pathogen‐derived components or host factors required for infection. The process is orchestrated by selective autophagy receptors that recognize specific cargoes for degradation. NEIGHBOUR OF BRCA1 (NBR1) functions as a receptor for autophagosomal degradation of ubiquitinated targets (Leong et al. 2022). Mechanistically, NBR1 can directly bind to viral particles, mediating their xenophagic degradation via recognition of ubiquitinated signals on these pathogens (Hafrén and Hofius 2017). In *Arabidopsis thaliana* , NBR1 (AtNBR1) is reported to directly target cauliflower mosaic virus (CaMV) capsid protein (CP) for degradation, limiting CaMV infection (Hafrén et al. 2017). NBR1‐mediated selective autophagy can suppress turnip mosaic virus (TuMV) infection by targeting viral RNA silencing suppressor HC‐Pro (Hafrén et al. 2018). Coronaviruses employ a conserved strategy of cleaving NBR1 via the 3C‐like protease NSP5 to evade host autophagy, emphasizing the importance of NBR1‐mediated xenophagy in antiviral defence (Li et al. 2025). Despite extensive evidence linking NBR1 to antiviral immunity, its role in plant–bacterium interactions remains largely unexplored. Only one report shows that the ubiquitinated effector XopL of *Xanthomonas campestris* pv. *vesicatoria* is degraded through NBR1/Joka2‐mediated selective autophagy (Leong et al. 2022). Whether NBR1 can target multiple effectors from the same bacterial pathogen is unknown. Here, we demonstrate that citrus NBR1 (CsNBR1) functions as a key immune regulator that promotes resistance to *C*Las by mediating degradation of its virulence effectors. This finding provides new mechanistic insight into citrus defence and offers a potential strategy for breeding HLB‐resistant cultivars.

To investigate the function of CsNBR1 in citrus immunity, we generated *CsNBR1* overexpression (OE‐1, OE‐2) and RNA interference (RNAi‐5, RNAi‐8) lines from epicotyl of Wanjincheng orange ( *Citrus sinensis* ) by an *Agrobacterium*‐mediated stable genetic transformation system (Peng et al. 2015), with CsNBR1 protein accumulation confirmed by western blotting (Figure S1). The NBR1 sequences are highly conserved in the *Citrus* genus (Figure S2a). NBR1 proteins are known positive regulators of antiviral defence (Hafrén et al. 2017; Hafrén et al. 2018). We next explored the defence pathways associated with CsNBR1 by comparing transcriptomic profiles between *CsNBR1*‐RNAi and wild‐type (WT) plants. Differentially expressed genes (DEGs) were identified using DESeq2 with thresholds of |log_2_fold change| ≥ 1 and false discovery rate (FDR) < 0.05. A total of 1324 downregulated genes and 965 upregulated genes in the *CsNBR1*‐RNAi lines compared with WT (Figure 1a). Gene Ontology enrichment revealed that DEGs in the ‘molecular function’ category were enriched for hydrolase activity, heme binding, and tetrapyrrole binding, suggesting that CsNBR1 influences multiple metabolic and defence processes (Figure 1b). KEGG pathway analysis showed that DEGs were mainly involved in plant hormone signal transduction (cit04075) and phenylpropanoid biosynthesis (cit00940) (Figure 1c), implying that CsNBR1 regulates hormonal and primary metabolic pathways. To validate the RNA‐seq results, the expression of four DEGs, including three downregulated genes, *WRKY22* (Cs_ont_5g003720), *AUX* (Cs_ont_5g004670) and *CPK10* (Cs_ont_5g047070), as well as one upregulated gene, *MPK3* (Cs_ont_8g004060). The results indicated that the expression patterns of all selected genes were consistent with those analysed by RNA‐seq (Figure 1d). To investigate the role of NBR1 in the immune response of citrus to *C*Las, transgenic citrus plants were graft‐inoculated using stems from *C*Las‐infected plants. At 4 months post‐inoculation (mpi), *CsNBR1*‐RNAi lines exhibited significantly higher *C*Las titres than WT, whereas no infection was detected in the *CsNBR1*‐OE lines. By 6 mpi, the *C*Las titre in *CsNBR1*‐OE lines increased slightly but remained markedly lower than in *CsNBR1*‐RNAi and WT plants (Figure 1e). At 8 mpi, *CsNBR1*‐RNAi plant leaves exhibited more pronounced *C*Las‐associated zinc deficiency and yellowing symptoms compared to WT, while *CsNBR1*‐OE lines exhibited mild symptoms (Figure 1f). These findings demonstrate that CsNBR1 confers enhanced resistance to *C*Las in citrus.

**FIGURE 1:**
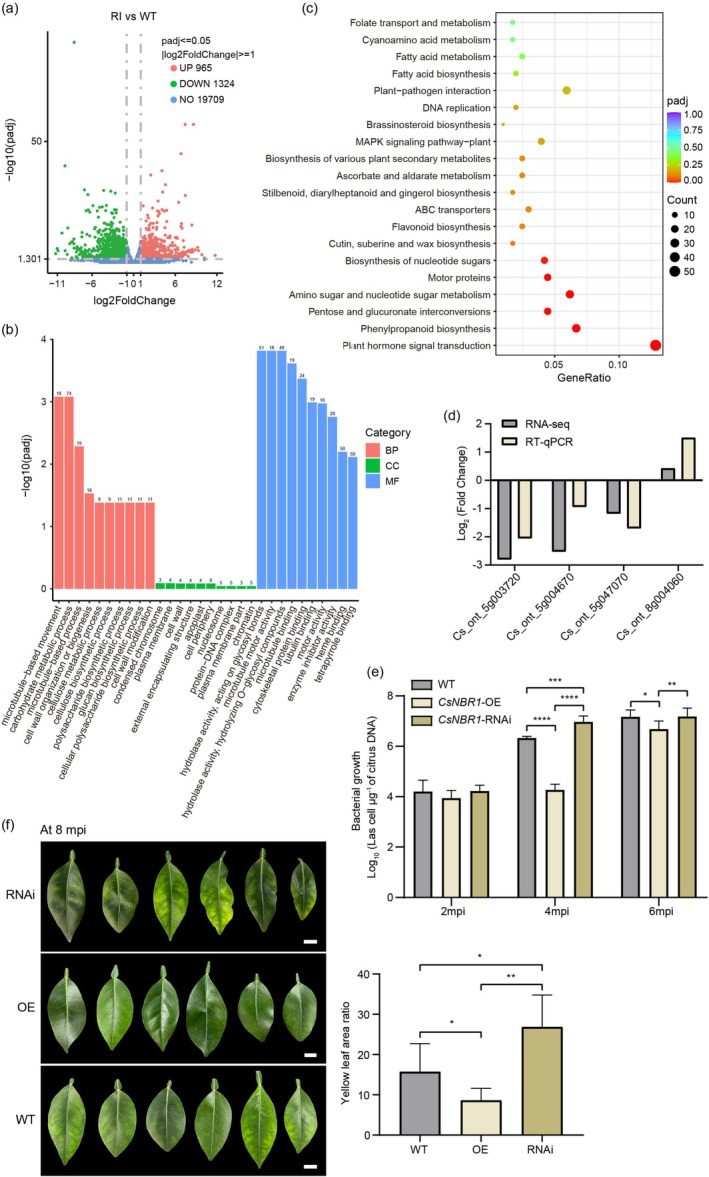
CsNBR1 positively regulates citrus disease resistance to ‘*Candidatus* Liberibacter asiaticus’ (*C*Las). (a) The volcano plot of differentially expressed genes (DEGs) between RNAi lines and wild type (WT). (b) Scatter plot of Gene Ontology (GO) enrichment analysis. The ordinate indicates the significantly enriched GO term. The abscissa represents −log_10_ (*p*_adj_) and gene count, respectively. (c) Scatter plot of KEGG enrichment. DEGs in *CsNBR1*‐RNAi and WT citrus plants were enriched in 20 KEGG pathways. (d) Validation of transcriptome data using reverse transcription‐quantitative PCR. *CsActin* gene was used as reference. (e) Quantitative analysis of *C*Las growth at 2, 4 and 6 months post‐infection (mpi) in *CsNBR1*‐OE (overexpression) or ‐RNAi citrus plants. Data represent the mean ± SD (*n* = 6). Statistical analysis was performed by two‐way ANOVA (*p < 0.05, **p < 0.01, ***p < 0.001, ****p < 0.0001). (f) Leaf symptoms and severity of WT and *CsNBR1* transgenic citrus at 8 mpi. Scale bar = 2 cm. The leaf yellowing area was quantified using ImageJ software. Statistical analysis was performed by Student's *t* test (*p < 0.05, **p < 0.01).

NBR1 recognizes and binds ubiquitinated protein complexes and interacts with ATG8 via a conserved interacting region (LIR) motif to promote selective autophagy (Hafrén et al. 2017; Zhou et al. 2021). To test whether CsNBR1 targets *C*Las‐secreted effectors, we performed a luciferase complementation imaging (LCI) assay in *Nicotiana benthamiana*. *C*Las effectors‐nLUC and CsNBR1‐cLUC were generated (all primers used in this study are listed in Table S1). Four previously identified virulence effector proteins, SDE5115, SDE1, SDE19 and SDE5, interacted with CsNBR1 (Figure 2a–d, Figure S3a) (Clark et al. 2018; Du et al. 2022; Huang et al. 2024; Zhao et al. 2025). Bimolecular fluorescence complementation (BiFC) results demonstrated that CsNBR1 associated with these effectors within cytoplasmic granules (Figure 2e, Figure S3b). A co‐immunoprecipitation (Co‐IP) assay was conducted to further validate these interactions in vivo, confirming the physical binding of CsNBR1 with SDE5115, SDE1, SDE19 and SDE5 (Figure 2f). Together, these results indicate that CsNBR1 interacts with the effector proteins SDE5115, SDE1, SDE19 and SDE5.

**FIGURE 2:**
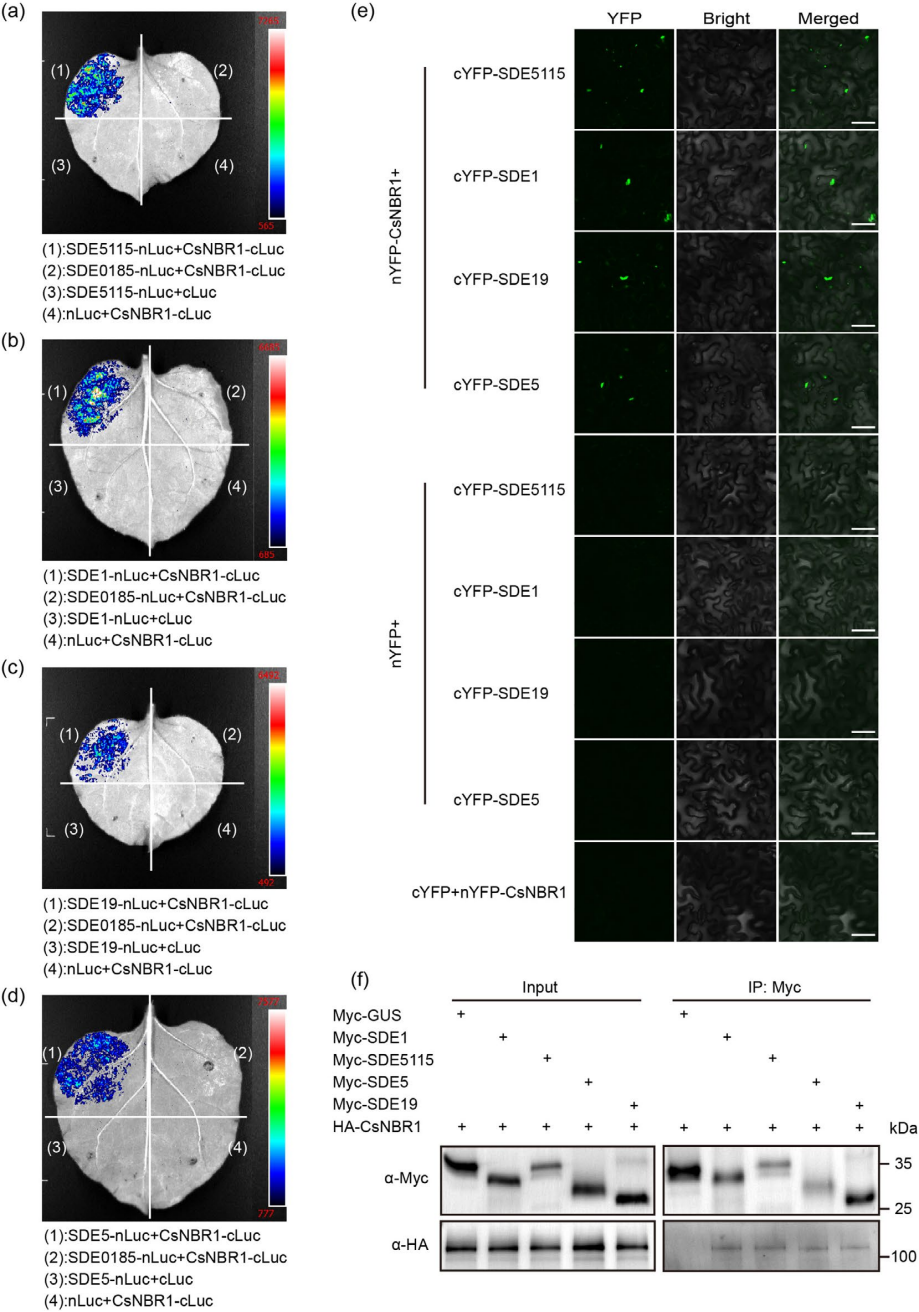
CsNBR1 interacts with ‘*Candidatus* Liberibacter asiaticus’ effectors SDE5115, SDE1, SDE19 and SDE5. (a–d) Results of the luciferase complementation imaging (LCI) assay show the interaction among CsNBR1 and SDE5115 (a), SDE1 (b), SDE19 (c), SDE5 (d) (without signal peptide). CsNBR1 was fused to the C‐terminus of cLUC. SDE5115, SDE1, SDE19 or SDE5 were fused to the N‐terminus of nLUC. Recombinant vectors were co‐expressed with cLUC‐CsNBR1 in *Nicotiana benthamiana* leaves. SDE0185‐nLUC + cLUC‐CsNBR1 was used as the negative control. (e) Results of the bimolecular fluorescence complementation (BiFC) assay show the interaction of CsNBR1 with SDE5115/SDE1/SDE19/SDE5 (without signal peptide). CsNBR1 was fused to the N‐terminus of nYFP. SDE5115, SDE1, SDE19, SDE5 were fused to the C‐terminus of cYFP. Fluorescence was observed and captured using an Olympus FV3000 confocal microscope at 2 days post‐inoculation (dpi). Scale bar = 50 μm. (f) Results of the co‐immunoprecipitation (Co‐IP) assay show the interaction of CsNBR1 with SDE5115/SDE1/SDE19/SDE5. HA‐CsNBR1 was co‐expressed with Myc‐SDE5115, SDE1, SDE19, SDE5 or Myc‐GUS (negative control) in *N. benthamiana* leaves. Total protein extracts were incubated with anti‐Myc beads at 4°C and detected with an anti‐HA antibody.

The ubiquitin (Ub)‐binding domain (UBA) of NBR1 is known to mediate selective binding to ubiquitin‐modified substrates (Xiang et al. 2024; Zhou et al. 2021). To determine the binding mechanism, we assessed whether the ubiquitin‐associated (UBA) domain of CsNBR1 mediates effector recognition. We performed LCI assays using the UBA domain of CsNBR1 (CsNBR1^UBA^) or CsNBR1 lacking the UBA domain (CsNBR1^ΔUBA^). The results showed that CsNBR1 physically interacted with SDE5115, SDE1, SDE19 and SDE5 specifically via its UBA domain (Figure S4). CsNBR1 shares high homology with the UBA domain of *N. benthamiana* NbNBR1 (Figure S2b). We further performed LCI assays by co‐expressing NbNBR1 with SDE5115, SDE1, SDE19 and SDE5 in *N. benthamiana* leaves, showing that NbNBR1 also interacts with these effectors, and that such interactions depend on the UBA domain (Figure S5). In addition, we transiently co‐expressed Myc‐SDE5115/SDE1/SDE19/SDE5 with FLAG‐tagged ubiquitin in *N. benthamiana* leaves and determined the ubiquitination of the immunoprecipitated SDE5115, SDE1, SDE19 and SDE5 protein by immunoblotting (Figure S6). Collectively, these results reveal that NBR1 specifically recognizes ubiquitinated SDE5115, SDE1, SDE19 and SDE5 via its UBA domain.

To investigate whether CsNBR1 degrades SDE5115, SDE1, SDE19 and SDE5 through the autophagic pathway, thereby positively regulating the immune response of citrus plants to *C*Las. In vivo protein degradation assays via agroinfiltration in 4‐week‐old *N. benthamiana* plants were performed. SDE5115, SDE1, SDE19 and SDE5 protein accumulation levels were reduced in leaves co‐expressing HA‐CsNBR1 (Figure 3a–d). Pretreatment of leaves with aspartic and cysteine proteases inhibitor E64d (100 μM) significantly inhibited the degradation of SDE5115, SDE1, SDE19 and SDE5 by HA‐CsNBR1, whereas proteasome inhibitor MG132 (50 μM) treatment failed to alter their stability (Figure 3a–d). Conversely, targeted silencing of the endogenous *NbNBR1* homologue via TRV‐mediated virus‐induced gene silencing (VIGS) resulted in a marked increase in SDE5115, SDE1, SDE19 and SDE5 protein levels compared to the TRV‐*GFP* control (Figure 3e). These data demonstrate that CsNBR1‐mediated selective autophagy targets and degrades multiple *C*Las effectors, thereby strengthening host resistance.

**FIGURE 3:**
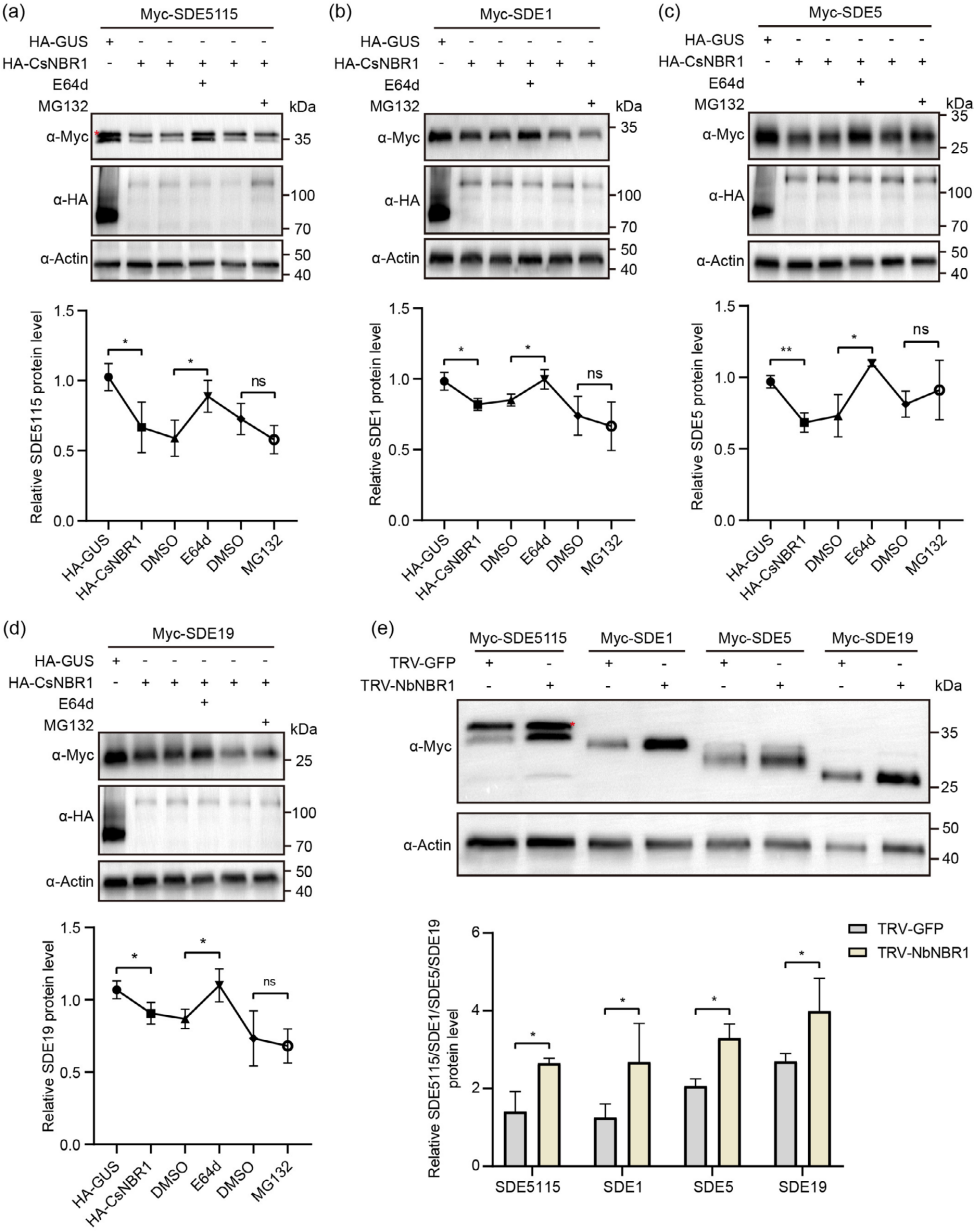
NBR1‐mediated selective autophagy degrades SDE5115, SDE1, SDE5 and SDE19. (a–d) Immunoblot analysing the effect of CsNBR1 on SDE5115 (a), SDE1 (b), SDE5 (c), and SDE19 (d) protein levels. HA‐CsNBR1 with Myc‐SDE5115, SDE1, SDE5, or SDE19 constructs were co‐expressed in *Nicotiana benthamiana*. Expression of HA‐GUS served as a control. Samples were treated with 100 μM E64d or 50 μM MG132, using an equivalent concentration of dimethyl sulphoxide (DMSO) as a control at 36 h post‐infiltration (hpi). Samples were collected at 48 hpi for total protein extraction. (e) Protein accumulation of SDE5115, SDE1, SDE5 and SDE19 in *N. benthamiana* plants silenced for *NbNBR1* (pTRV‐*NbNBR1*) compared with control plants (pTRV‐*GFP*). (a–e) Myc‐SDE5115, SDE1, SDE5, SDE19 protein levels were detected using an anti‐Myc antibody. Expression of CsNBR1 protein was verified using an anti‐HA antibody. Actin antibody serves as a loading control. Relative protein abundance was quantified in ImageJ software. Data represent mean ± SD of three biological replicates (*p < 0.05, **p < 0.01, Student's *t* test).

To further elucidate the immune function of CsNBR1, we used SDE1 as a model effector, as previous work showed that the potato virus X (PVX)‐mediated expression of SDE1 in *N. benthamiana* induces dwarfing and leaf curling (Shen et al. 2022). Transient co‐expression experiments with PVX‐FLAG‐SDE1 (positive control) with either PVX empty vector (PVX‐EV) or PVX‐FLAG‐CsNBR1 in 4‐week‐old *N. benthamiana* plants were performed. The result showed that at 12 days post‐inoculation (dpi), the systemic leaves expressing PVX‐FLAG‐SDE1 alone or co‐expressing with PVX‐EV exhibited severe curling, whereas those co‐expressing with PVX‐FLAG‐CsNBR1 displayed only mild symptoms (Figure 4a,b). Statistical analysis revealed significantly greater plant height in the latter group at 12 and 18 dpi (Figure 4c). Notably, expression of PVX‐EV or PVX‐FLAG‐CsNBR1 alone had no effect on the growth of *N. benthamiana* (Figure S7). Reverse transcription‐quantitative PCR (RT‐qPCR) and western blot analyses demonstrated reduced SDE1 protein levels in the PVX‐FLAG‐SDE1 + PVX‐FLAG‐CsNBR1 group at 12 dpi (Figure 4d). Furthermore, inoculation of *Pseudomonas syringae* pv. *tomato* (Pst) DC3000 into the systemic leaves of these group plants resulted in milder leaf blight symptoms and fewer colonies in the PVX‐FLAG‐SDE1 + PVX‐FLAG‐CsNBR1 group at 3 dpi (Figure S8). These findings indicate that CsNBR1 inhibits SDE1‐mediated immune suppression, enhances autophagic degradation of SDE1, and promotes disease resistance.

**FIGURE 4:**
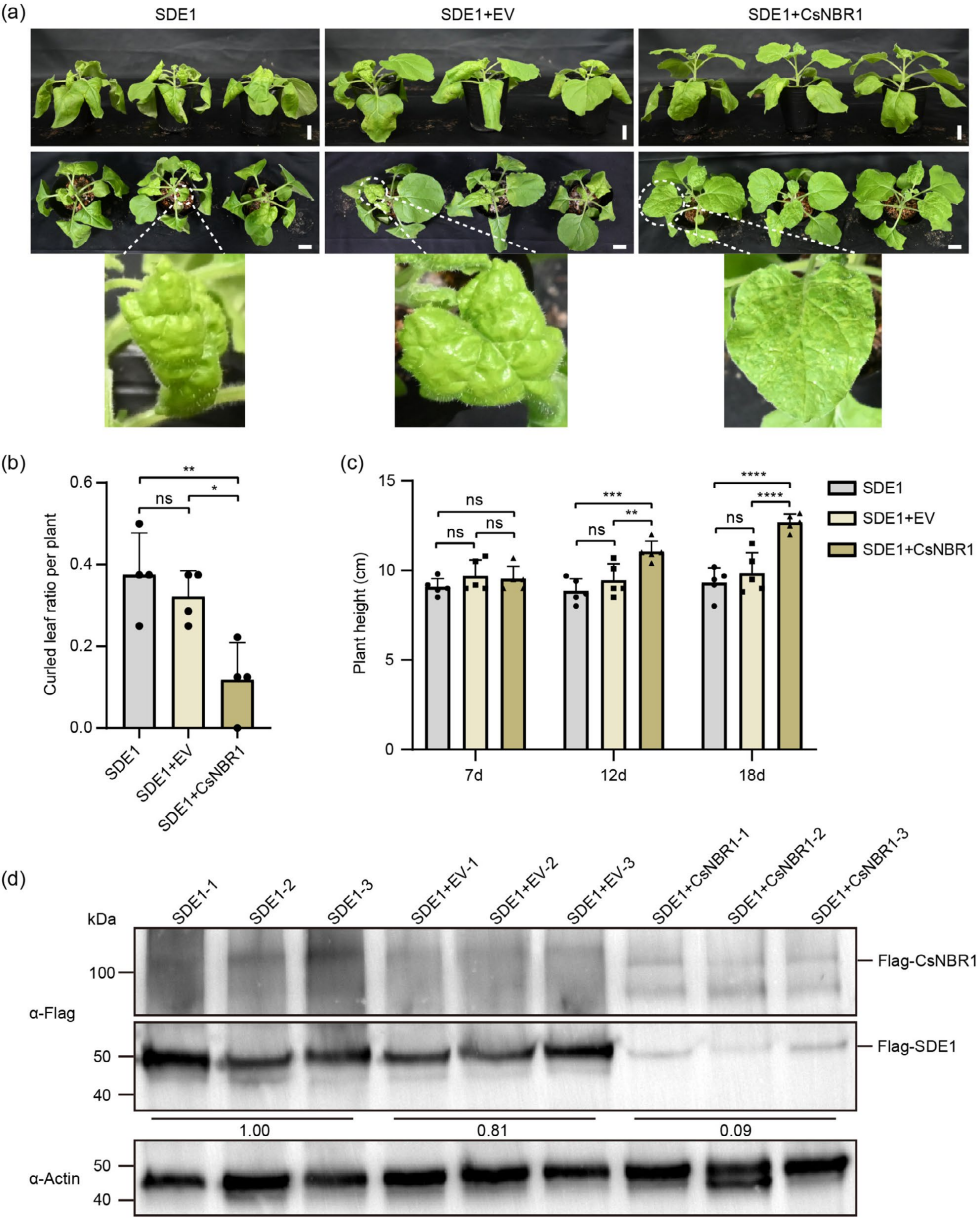
The effect of CsNBR1 on the pathogenicity of heterologous PVX‐FLAG‐SDE1. (a) Symptoms induced in *Nicotiana benthamiana* plants by inoculation of PVX‐FLAG‐SDE1 (positive control), PVX‐FLAG‐SDE1 + PVX‐EV (PVX empty vector, negative control), PVX‐FLAG‐SDE1 + PVX‐FLAG‐CsNBR1 at 12 days post‐infiltration (dpi). Scale bar = 2 cm. (b) Ratio of curled leaf number to total leaf number per plant at 12 dpi. Statistical analysis was performed by one‐way ANOVA (*p < 0.05, **p < 0.01). (c) Height induced in *N. benthamiana* plants by inoculation of PVX‐FLAG‐SDE1, PVX‐FLAG‐SDE1 + PVX‐EV, PVX‐FLAG‐SDE1 + PVX‐FLAG‐CsNBR1 at 7, 12 and 18 dpi. Data represent the mean ± SD (*n* = 4). Statistical analysis was performed by two‐way ANOVA (**p < 0.01, ***p < 0.001, ****p < 0.0001). (d) Western blotting analysis of SDE1 accumulation with anti‐FLAG antibody in systemic leaves of *N. benthamiana* plants at 12 dpi. Actin antibody was used as an equal loading control.

Although NBR1 is known to mediate defence against various viruses and a few bacteria by facilitating the autophagic degradation of virulence factors (Hafrén et al. 2017; Hafrén et al. 2018; Leong et al. 2022), its role in combating *C*Las was previously unclear. Currently, the inability to culture *C*Las in vitro and its strict phloem tropism have impeded research into its pathogenicity and control (Lewis et al. 2022). To date, transgenic citrus overexpressing *Arabidopsis NPR1* (*AtNPR1*), *NPR1‐like* (*CiNPR4*) and *CsSAMT1* have been shown to exhibit HLB tolerance (Dutt et al. 2015; Peng et al. 2021; Zou et al. 2021). We recently reported that overexpressing a dominant‐negative U‐box E3 ligase PUB21 (PUB21DN) stabilizes MYC2, conferring HLB resistance (Zhao et al. 2025). In this study, our transcriptome and transgenic resistance evaluation results reveal that CsNBR1 modulates plant hormone signalling and primary metabolism to enhance resistance to HLB. At the protein level, NBR1 interacts with ubiquitinated SDE5115, SDE1, SDE19, SDE5 via its UBA domain known to bind ubiquitinated substrates (Xiang et al. 2024) and degrades them via autophagy, which is consistent with NBR1's known role in degrading CaMV CP, TuMV HC‐Pro and XopL of *X. campestris* pv. *vesicatoria* (Hafrén et al. 2017, 2018; Leong et al. 2022). These results indicate that CsNBR1 is a crucial molecular target for breeding resistant varieties. The UBA domain of CsNBR1 recognizes ubiquitinated SDE5115, SDE1, SDE19 and SDE5 and mediates their autophagic degradation, verifying the selective autophagy function of CsNBR1.

NBR1 suppresses SDE1‐induced *N. benthamiana* plant dwarfism and leaf curling by reducing SDE1 protein levels (Shen et al. 2022), thereby enhancing resistance to Pst DC3000. This suggests NBR1's functional conservation and potential role in defending against other citrus pathogens. Collectively, our work advances understanding of NBR1 in plant defence and provides a molecular basis for HLB resistance. Building on the insights from this study, future development of citrus germplasm with enhanced HLB resistance or tolerance via genetic engineering of resistance genes (e.g., *CsNBR1*) will offer sustainable solutions to address the challenge of HLB control.

## Author Contributions

**Xuejin Cui:** conceptualization, validation, methodology, investigation. **Fang Fang:** software, formal analysis. **Zaiyu Yang:** software, formal analysis. **Shimian Ma:** software, formal analysis. **Yaqian Shi:** data curation, writing – original draft, methodology, validation, conceptualization, visualization. **Changyong Zhou:** supervision, resources. **Yalin Mei:** software, formal analysis. **Xuefeng Wang:** project administration, funding acquisition, writing – review and editing.

## Funding

This work was supported by grants from National Natural Sciences Foundation of China (U23A20196), Fundamental and Interdisciplinary Disciplines Breakthrough Plan of the Ministry of Education of China (JYB2025XDXM701), Special Fund for Youth Team of Southwest University (SWU‐XJLJ202310), Innovation Research 2035 Pilot Plan of Southwest University (SWU‐XDZD22002) and Southwest University research and innovation project (SWUB24080).

## Conflicts of Interest

The authors declare no conflicts of interest.

## Supporting information


**Figure S1:** Positive identification of *CsNBR1* transgenic citrus plants. (a, b) Diagram of the construction of *CsNBR1*‐OE (overexpression) (a) or ‐RNAi (b) recombinant expression vector. (c) Phenotypic analyses of *CsNBR1*‐OE plants. (d) Phenotypic analyses of *CsNBR1*‐RNAi plants. Under blue light, transgenic plants displayed green fluorescence, whereas wild‐type (WT) citrus emitted red fluorescence. (e) Reverse transcription‐quantitative PCR analysing the mRNA levels of *CsNBR1* in *CsNBR1*‐OE or ‐RNAi citrus plants. *CsActin* served as an internal reference. Data represent the mean ± SD of three biological replicates. Statistical analysis was performed by one‐way ANOVA (**p < 0.01, ****p < 0.0001). (f) Immunoblot analysis of protein levels of CsNBR1 in *CsNBR1*‐OE citrus plants. The total protein was extracted and subjected to immunoblotting with anti‐NBR1 and anti‐FLAG antibodies. Actin served as a loading control.

**Figure S2:** Bioinformatic analysis of CsNBR1. (a) Phylogenetic tree analysis of different species NBR1. The phylogenetic tree was generated by MEGA X software using a neighbour‐joining method with the 1000‐replicate bootstrap. (b) Multiple sequence alignment analysis of CsNBR1 and NbNBR1 by DNAMAN 6.0. The red line indicates ubiquitin‐associated (UBA) domain.

**Figure S3:** SDE0185 cannot interact with CsNBR1. (a) Luciferase complementation imaging (LCI) assays to test the interaction between SDE0185 and CsNBR1 in *Nicotiana benthamiana* leaves. SDE0185 (without signal peptide) were fused to the N‐terminal of LUC. (b) Bimolecular fluorescence complementation (BiFC) assays to test the interaction between SDE0185 and CsNBR1 in *N. benthamiana* leaves. SDE0185 was fused to the C‐terminus of cYFP. Scale bar = 100 μm.

**Figure S4:** The UBA domain of CsNBR1 interacts with SDE5115, SDE1, SDE19 and SDE5. (a–d) Luciferase complementation imaging (LCI) assays to test the interaction among SDE5115 (a), SDE1 (b), SDE19 (c), SDE5 (d), CsNBR1^UBA^ and CsNBR1^ΔUBA^ in *Nicotiana benthamiana* leaves. SDE5115, SDE1, SDE19, SDE5 (without signal peptide) were fused to the N‐terminal of LUC. CsNBR1^UBA^ and CsNBR1^ΔUBA^ were fused to the C‐terminal portion of LUC, respectively. *Agrobacterium tumefaciens* GV3101 carrying indicated vectors was co‐infiltrated into *N. benthamiana* leaves. Pictures were captured by using a CCD imaging apparatus 48 h post‐infiltration.

**Figure S5:** The UBA domain of NbNBR1 interacts with SDE5115, SDE1, SDE19, and SDE5. (a–d) Luciferase complementation imaging (LCI) assays to test the interaction among SDE5115 (a), SDE1 (b), SDE19 (c), SDE5 (d), NbNBR1 in *Nicotiana benthamiana* leaves. SDE5115, SDE1, SDE19, SDE5 (without signal peptide) were fused to the N‐terminal of LUC. NbNBR1 were fused to the C‐terminal portion of LUC, respectively. (e–h) LCI assays to test the interaction among SDE5115 (e), SDE1 (f), SDE19 (g), SDE5 (h), NbNBR1^UBA^ and NbNBR1^ΔUBA^ in *N. benthamiana* leaves. NbNBR1^UBA^ and NbNBR1^ΔUBA^ were fused to the C‐terminal portion of LUC, respectively. Pictures were captured by using a CCD imaging apparatus 48 h post‐infiltration.

**Figure S6:** SDE1, SDE5115, SDE5, and SDE19 can be ubiquitinated in planta. Total proteins from *Nicotiana benthamiana* leaves expressing Myc‐SDE1/SDE5115/SDE5/SDE19 or Myc‐GUS with FLAG‐Ub were extracted, followed by immunoprecipitation with anti‐FLAG beads. Myc‐GUS served as a negative control.

**Figure S7:** The effect of PVX‐CsNBR1 on the growth of *Nicotiana benthamiana* plants. (a) Symptoms induced in *N. benthamiana* plants by inoculation with PVX‐EV (empty vector), PVX‐FLAG‐CsNBR1 at 12 days post‐inoculation (dpi). Scale bar = 2 cm. (b) Western blotting analysis of CsNBR1 accumulation with an anti‐FLAG antibody in systemic leaves of *N. benthamiana* plants at 12 dpi. Actin antibody was used as an equal loading control.

**Figure S8:** The effect of the interaction between CsNBR1 and SDE1 on the immunity of *Nicotiana benthamiana*. (a) Symptoms of *N. benthamiana* leaves infected by *Pseudomonas syringae* pv. *tomato* (Pst) DC3000. Pst DC3000 was infiltrated into systemic leaves of *N. benthamiana* expressing PVX‐FLAG‐SDE1, PVX‐FLAG‐SDE1 + PVX‐EV, and PVX‐FLAG‐SDE1 + PVX‐FLAG‐CsNBR1. Leaf symptoms were observed at 3 days post‐inoculation (dpi). (b) Statistics of Pst DC3000 colony numbers. 10 μL of sap from samples diluted 10^4^, 10^5^ and 10^6^ times was spread on King's B (KB) solid medium, cultured at 28°C for 2 days, and the number of colonies was counted to calculate the bacterial load per square centimetre of leaf. Data represent the mean ± SD (*n* = 3). Statistical analysis was performed by one‐way ANOVA (**p < 0.01, **p < 0.001).

**Table S1:** List of primers used in this study.

## Data Availability

The data used to support the findings of this study are available from the corresponding author upon request.
