# Supplementary figures and images for: Selective Autophagy Receptor CsNBR1 Confers Citrus Huanglongbing Resistance by Degrading ‘Candidatus Liberibacter Asiaticus’ Virulence Effectors
Source: Mol Plant Pathol. 2026 Jun 29;27(7):e70310. doi: 10.1111/mpp.70310 (PMC13315814; doi:10.1111/mpp.70310)

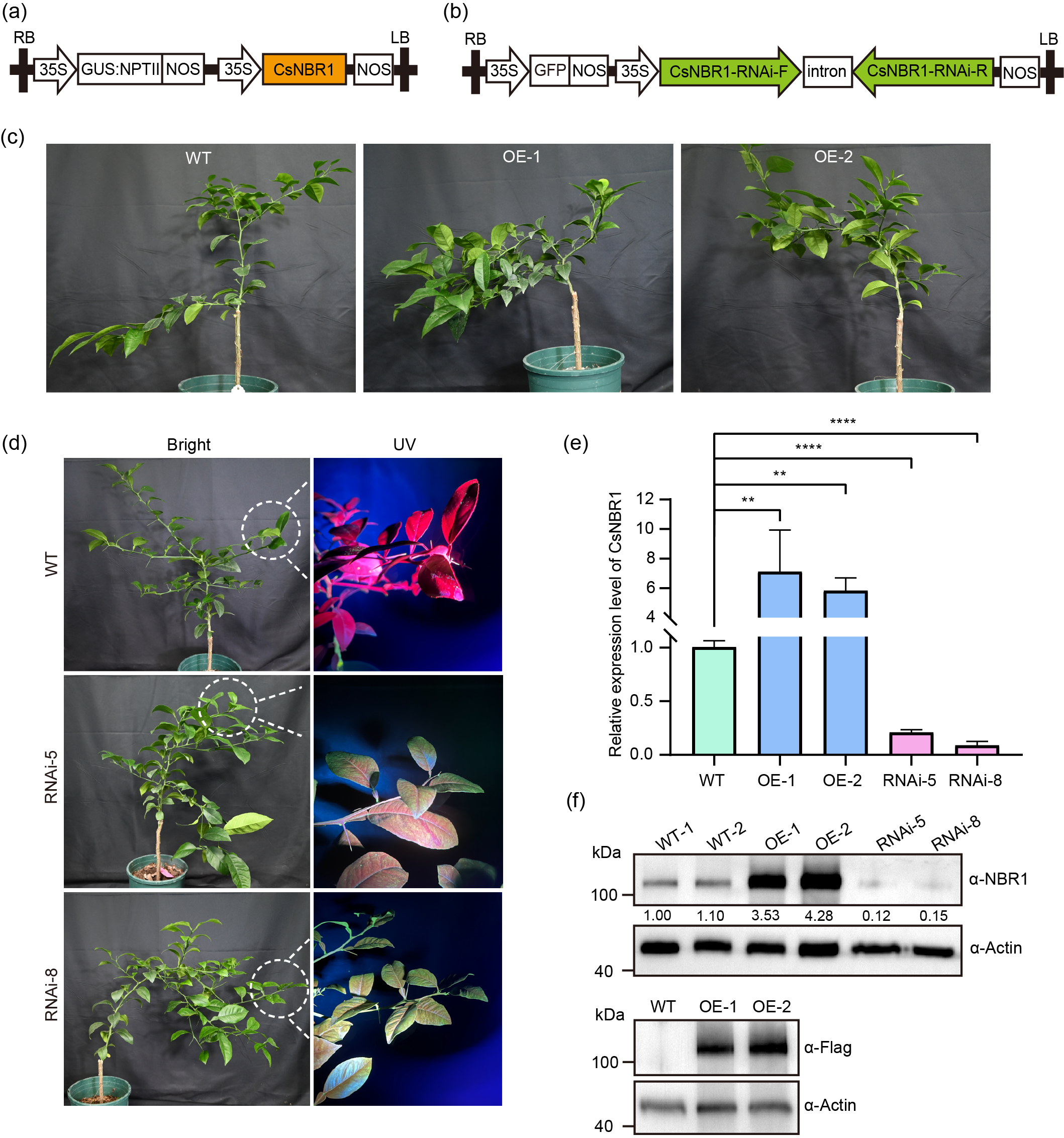

Supplement: Supplementary file 1 — Figure S1: Positive identification of CsNBR1 transgenic citrus plants. (a, b) Diagram of the construction of CsNBR1‐OE (overexpression) (a) or ‐RNAi (b) recombinant expression vector. (c) Phenotypic analyses of CsNBR1‐OE plants. (d) Phenotypic analyses of CsNBR1‐RNAi plants. Under blue light, transgenic plants displayed green fluorescence, whereas wild‐type (WT) citrus emitted red fluorescence. (e) Reverse transcription‐quantitative PCR analysing the mRNA levels of CsNBR1 in CsNBR1‐OE or ‐RNAi citrus plants. CsActin served as an internal reference. Data represent the mean ± SD of three biological replicates. Statistical analysis was performed by one‐way ANOVA (**p < 0.01, ****p < 0.0001). (f) Immunoblot analysis of protein levels of CsNBR1 in CsNBR1‐OE citrus plants. The total protein was extracted and subjected to immunoblotting with anti‐NBR1 and anti‐FLAG antibodies. Actin served as a loading control. [file MPP-27-e70310-s001.tif]

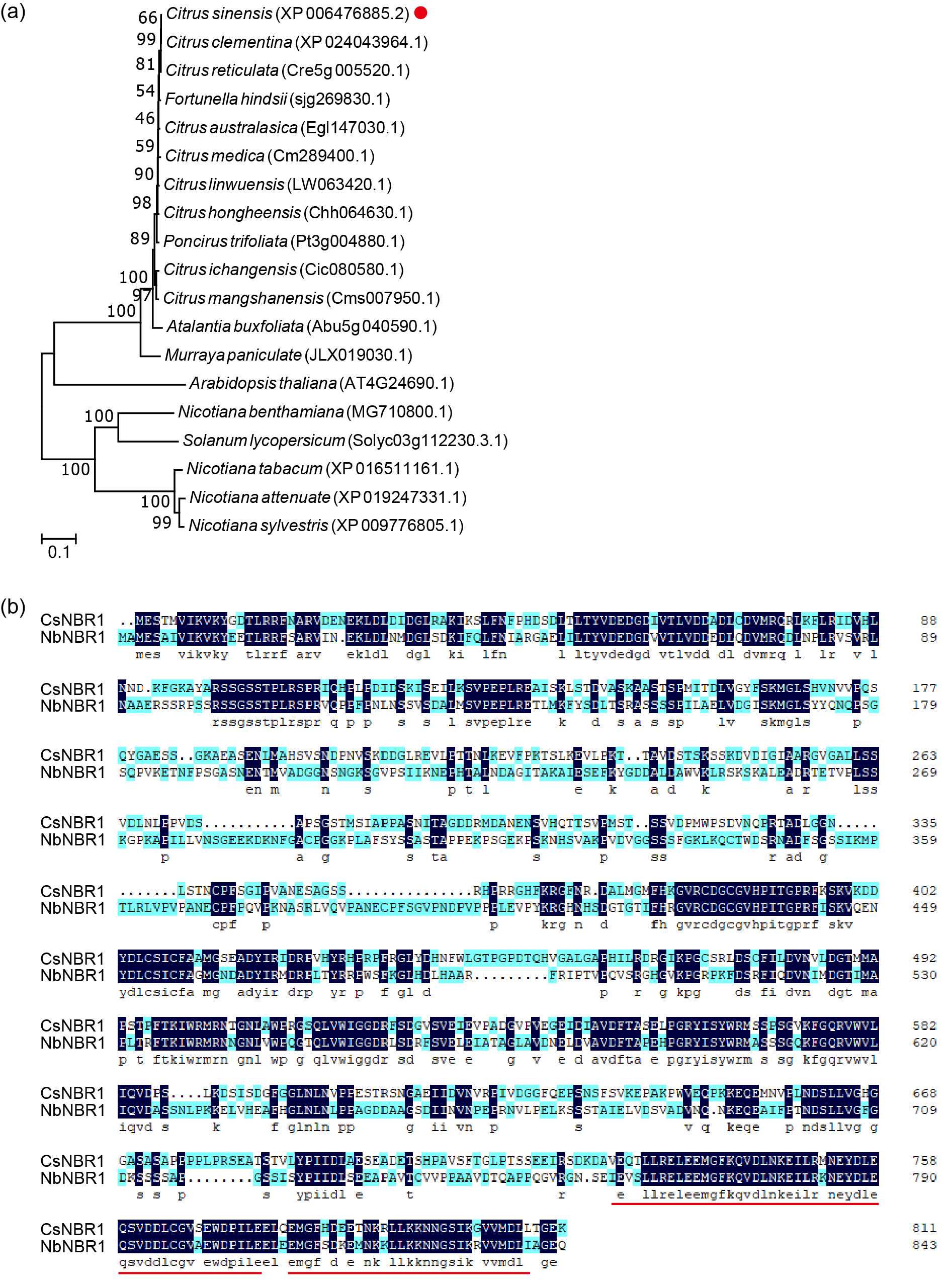

Supplement: Supplementary file 2 — Figure S2: Bioinformatic analysis of CsNBR1. (a) Phylogenetic tree analysis of different species NBR1. The phylogenetic tree was generated by MEGA X software using a neighbour‐joining method with the 1000‐replicate bootstrap. (b) Multiple sequence alignment analysis of CsNBR1 and NbNBR1 by DNAMAN 6.0. The red line indicates ubiquitin‐associated (UBA) domain. [file MPP-27-e70310-s008.tif]

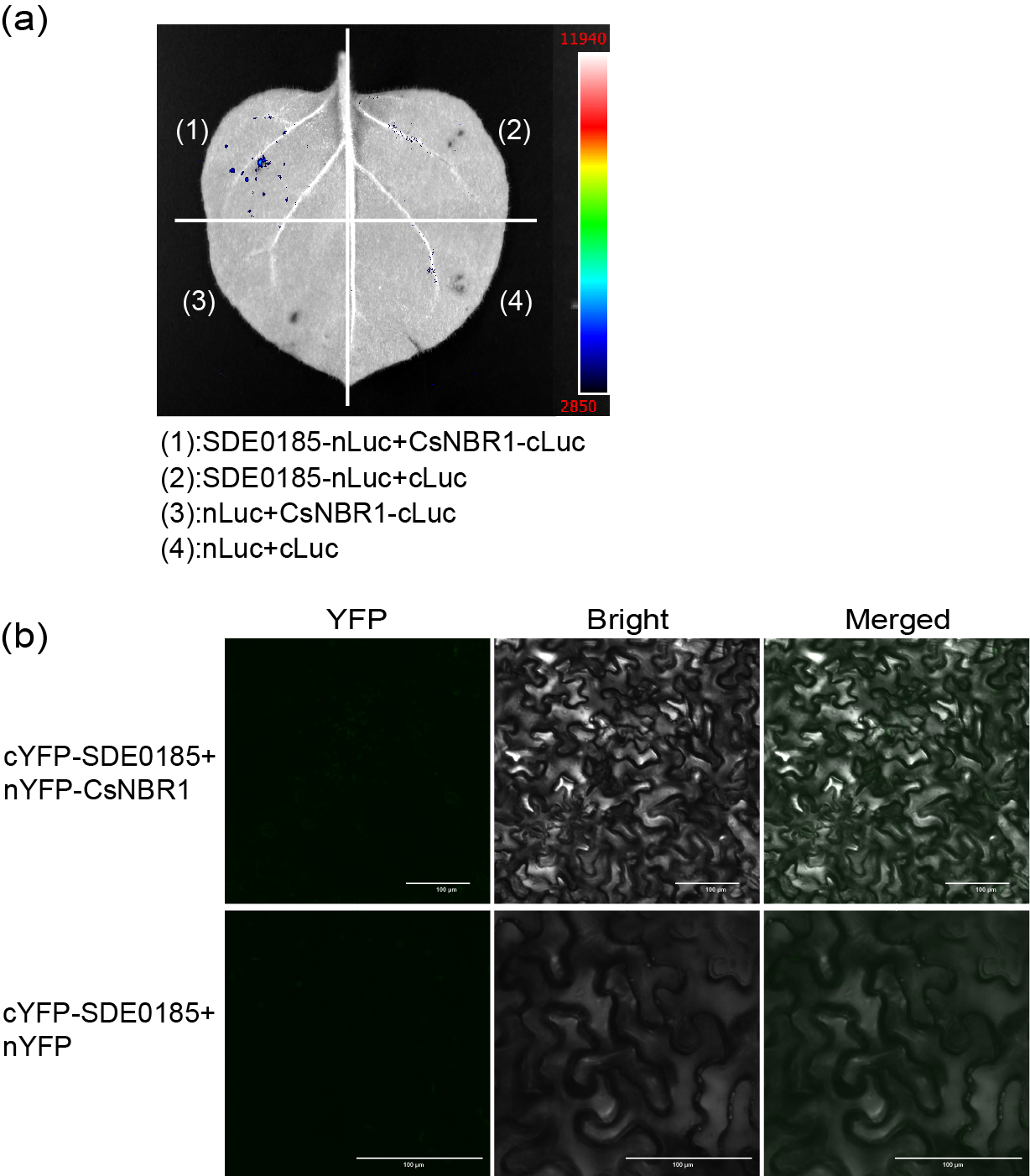

Supplement: Supplementary file 3 — Figure S3: SDE0185 cannot interact with CsNBR1. (a) Luciferase complementation imaging (LCI) assays to test the interaction between SDE0185 and CsNBR1 in Nicotiana benthamiana leaves. SDE0185 (without signal peptide) were fused to the N‐terminal of LUC. (b) Bimolecular fluorescence complementation (BiFC) assays to test the interaction between SDE0185 and CsNBR1 in N. benthamiana leaves. SDE0185 was fused to the C‐terminus of cYFP. Scale bar = 100 μm. [file MPP-27-e70310-s005.tif]

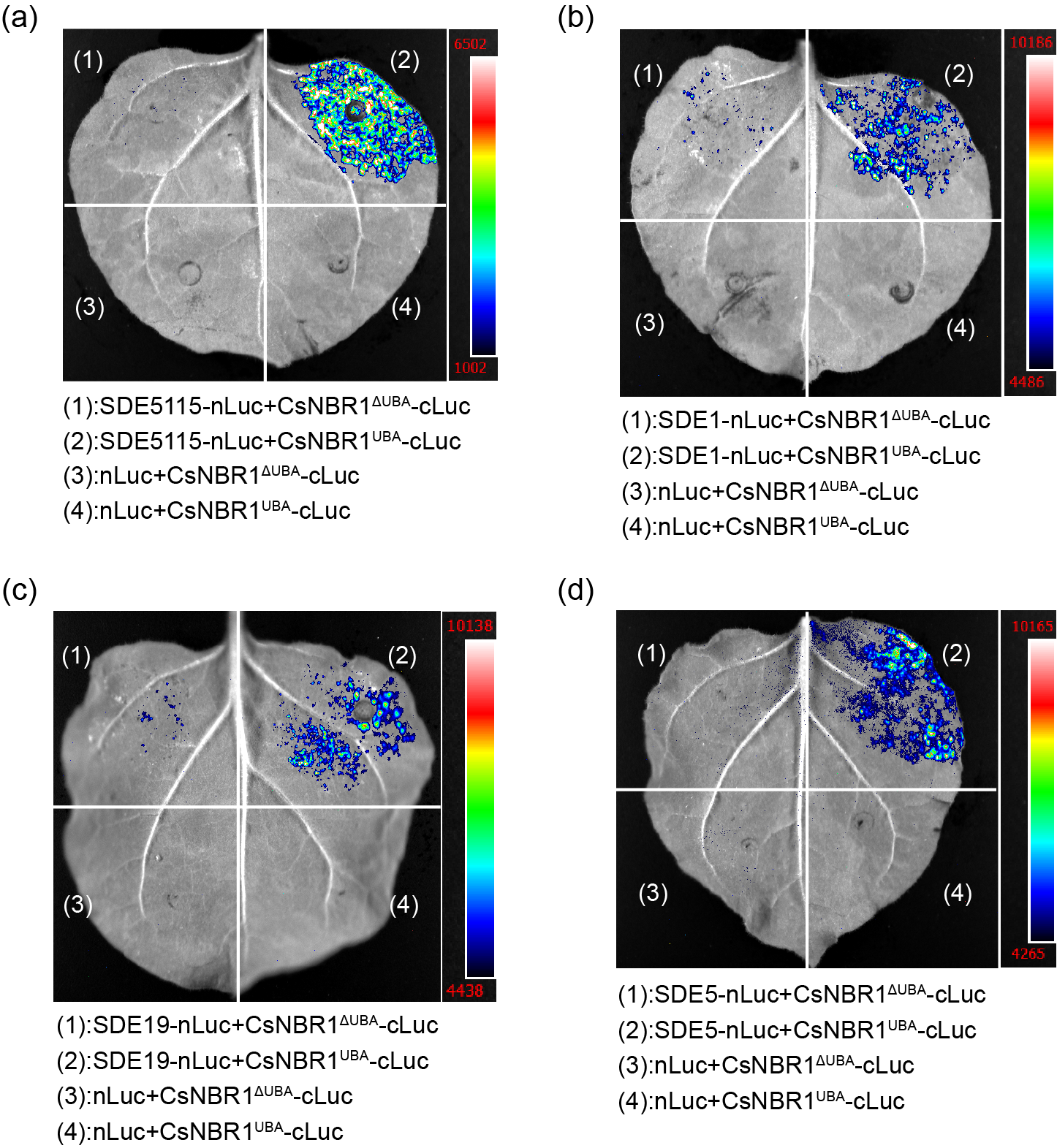

Supplement: Supplementary file 4 — Figure S4: The UBA domain of CsNBR1 interacts with SDE5115, SDE1, SDE19 and SDE5. (a–d) Luciferase complementation imaging (LCI) assays to test the interaction among SDE5115 (a), SDE1 (b), SDE19 (c), SDE5 (d), CsNBR1UBA and CsNBR1ΔUBA in Nicotiana benthamiana leaves. SDE5115, SDE1, SDE19, SDE5 (without signal peptide) were fused to the N‐terminal of LUC. CsNBR1UBA and CsNBR1ΔUBA were fused to the C‐terminal portion of LUC, respectively. Agrobacterium tumefaciens GV3101 carrying indicated vectors was co‐infiltrated into N. benthamiana leaves. Pictures were captured by using a CCD imaging apparatus 48 h post‐infiltration. [file MPP-27-e70310-s003.tif]

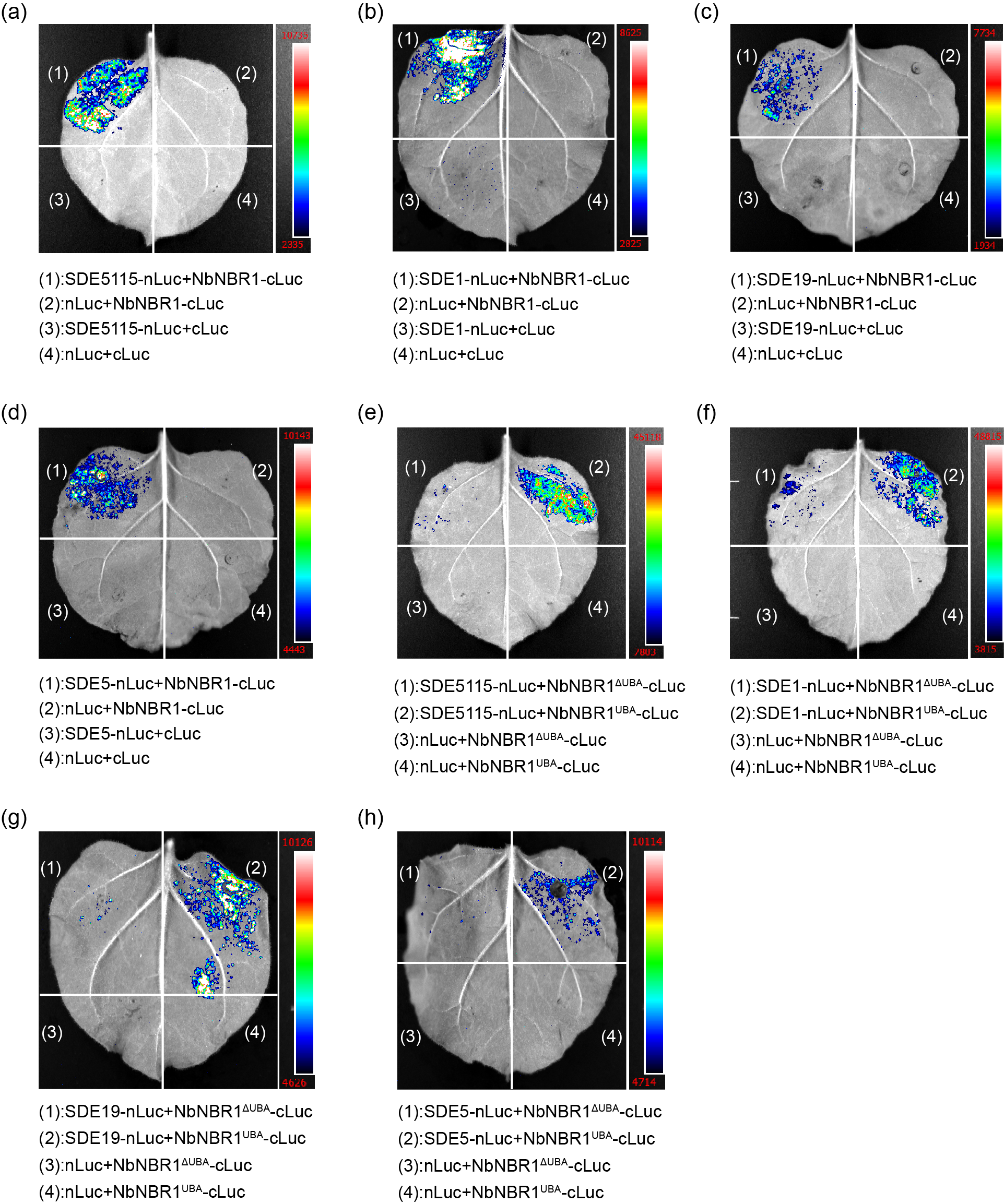

Supplement: Supplementary file 5 — Figure S5: The UBA domain of NbNBR1 interacts with SDE5115, SDE1, SDE19, and SDE5. (a–d) Luciferase complementation imaging (LCI) assays to test the interaction among SDE5115 (a), SDE1 (b), SDE19 (c), SDE5 (d), NbNBR1 in Nicotiana benthamiana leaves. SDE5115, SDE1, SDE19, SDE5 (without signal peptide) were fused to the N‐terminal of LUC. NbNBR1 were fused to the C‐terminal portion of LUC, respectively. (e–h) LCI assays to test the interaction among SDE5115 (e), SDE1 (f), SDE19 (g), SDE5 (h), NbNBR1UBA and NbNBR1ΔUBA in N. benthamiana leaves. NbNBR1UBA and NbNBR1ΔUBA were fused to the C‐terminal portion of LUC, respectively. Pictures were captured by using a CCD imaging apparatus 48 h post‐infiltration. [file MPP-27-e70310-s006.tif]

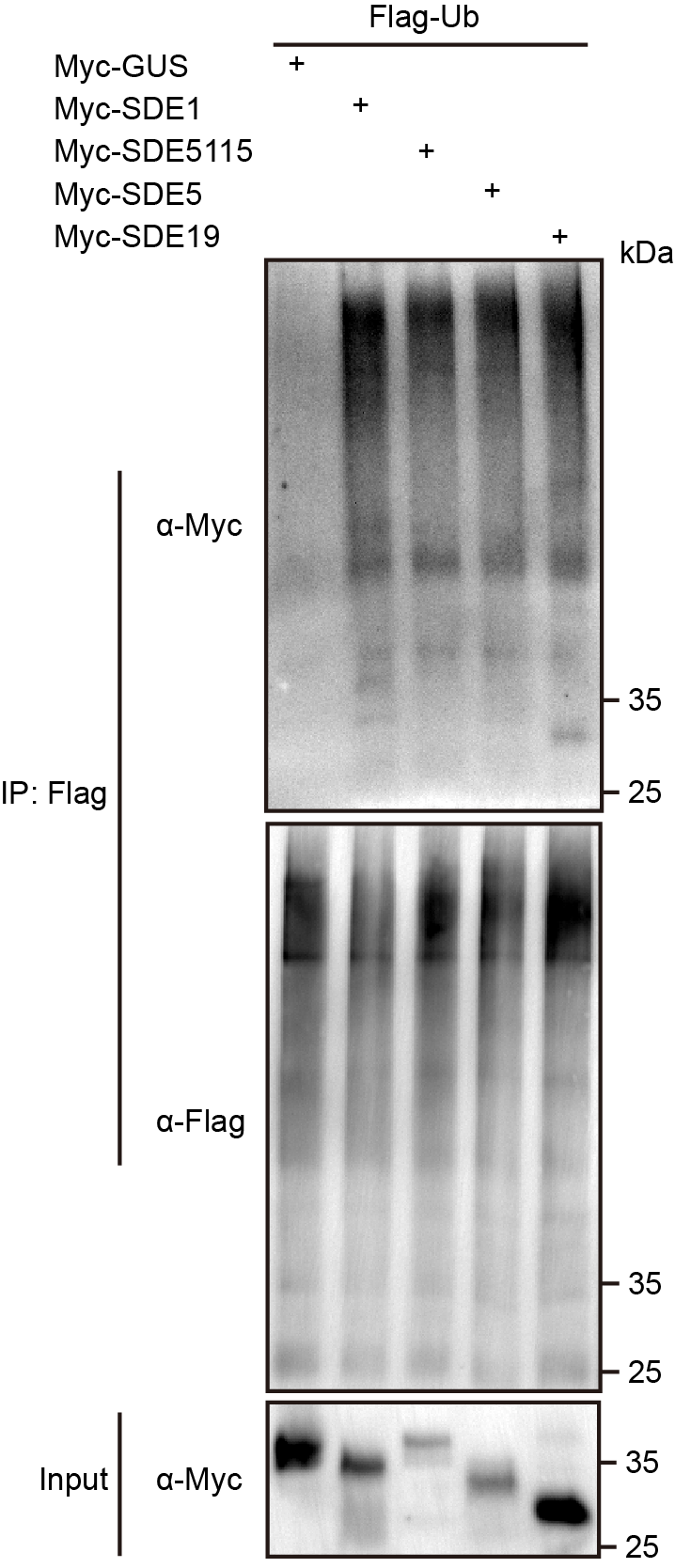

Supplement: Supplementary file 6 — Figure S6: SDE1, SDE5115, SDE5, and SDE19 can be ubiquitinated in planta. Total proteins from Nicotiana benthamiana leaves expressing Myc‐SDE1/SDE5115/SDE5/SDE19 or Myc‐GUS with FLAG‐Ub were extracted, followed by immunoprecipitation with anti‐FLAG beads. Myc‐GUS served as a negative control. [file MPP-27-e70310-s009.tif]

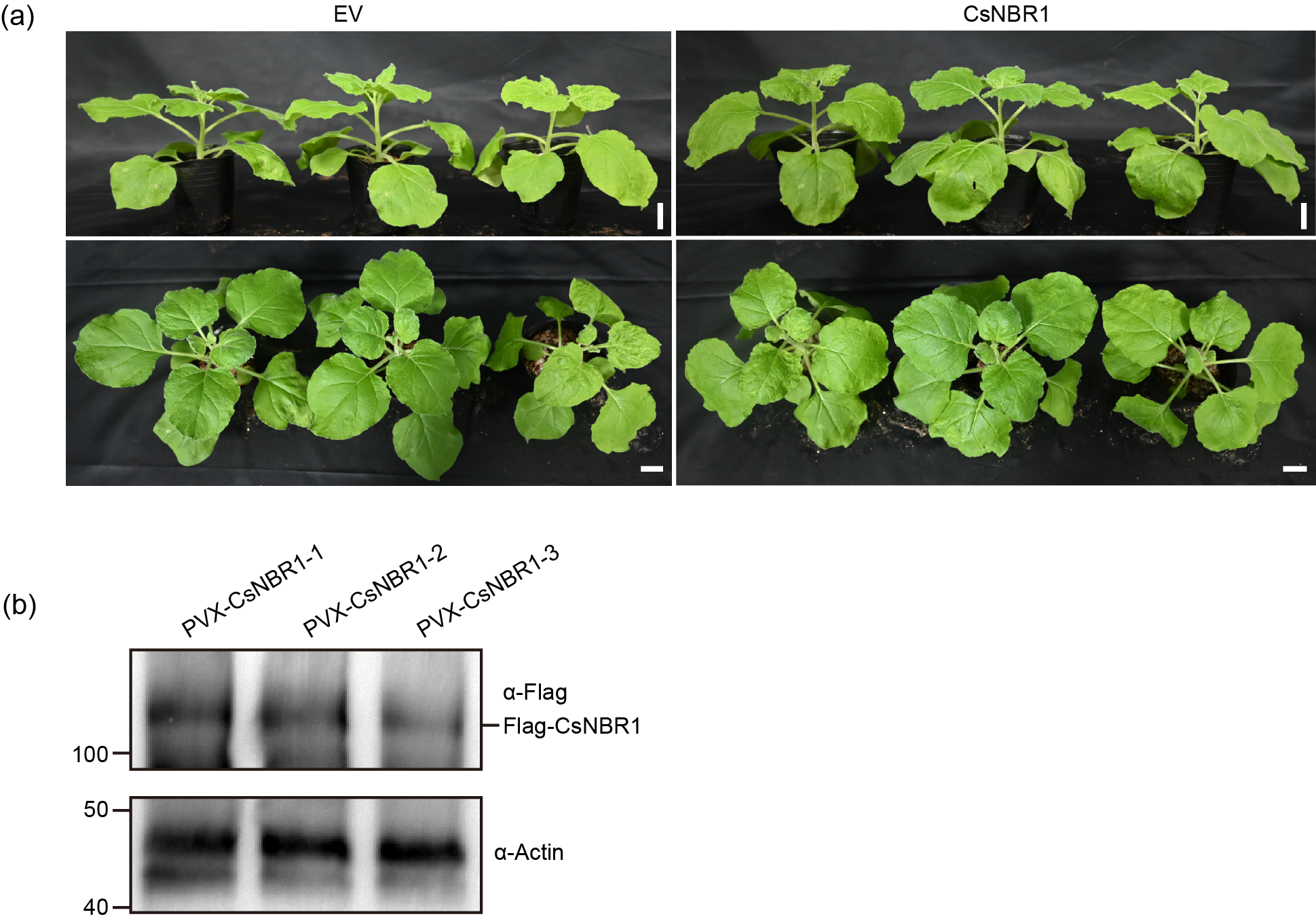

Supplement: Supplementary file 7 — Figure S7: The effect of PVX‐CsNBR1 on the growth of Nicotiana benthamiana plants. (a) Symptoms induced in N. benthamiana plants by inoculation with PVX‐EV (empty vector), PVX‐FLAG‐CsNBR1 at 12 days post‐inoculation (dpi). Scale bar = 2 cm. (b) Western blotting analysis of CsNBR1 accumulation with an anti‐FLAG antibody in systemic leaves of N. benthamiana plants at 12 dpi. Actin antibody was used as an equal loading control. [file MPP-27-e70310-s002.tif]

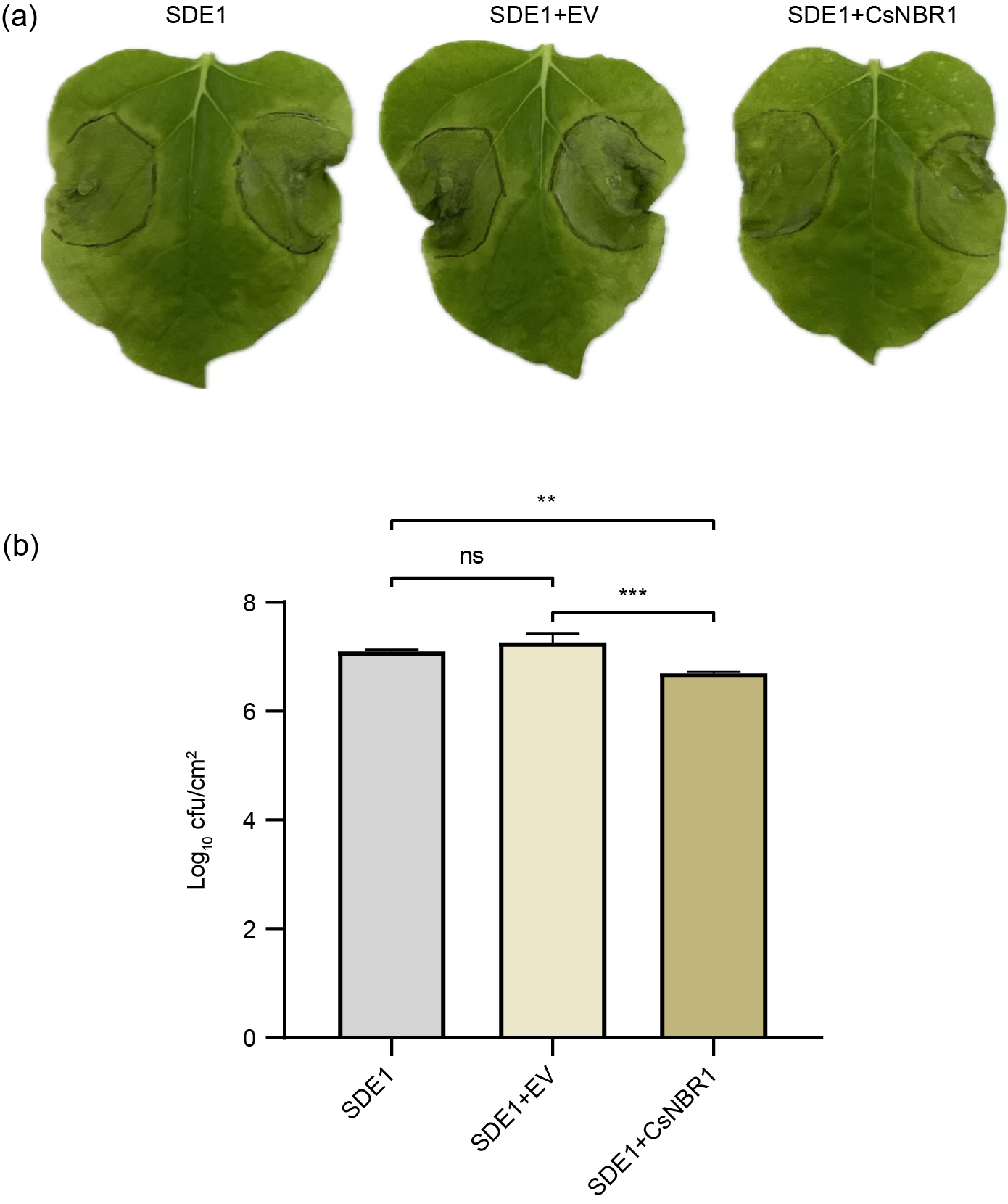

Supplement: Supplementary file 8 — Figure S8: The effect of the interaction between CsNBR1 and SDE1 on the immunity of Nicotiana benthamiana. (a) Symptoms of N. benthamiana leaves infected by Pseudomonas syringae pv. tomato (Pst) DC3000. Pst DC3000 was infiltrated into systemic leaves of N. benthamiana expressing PVX‐FLAG‐SDE1, PVX‐FLAG‐SDE1 + PVX‐EV, and PVX‐FLAG‐SDE1 + PVX‐FLAG‐CsNBR1. Leaf symptoms were observed at 3 days post‐inoculation (dpi). (b) Statistics of Pst DC3000 colony numbers. 10 μL of sap from samples diluted 104, 105 and 106 times was spread on King's B (KB) solid medium, cultured at 28°C for 2 days, and the number of colonies was counted to calculate the bacterial load per square centimetre of leaf. Data represent the mean ± SD (n = 3). Statistical analysis was performed by one‐way ANOVA (**p < 0.01, **p < 0.001). [file MPP-27-e70310-s004.tif]
